# Editorial: Microbial Exopolysaccharides: From Genes to Applications

**DOI:** 10.3389/fmicb.2016.00308

**Published:** 2016-03-11

**Authors:** Jochen Schmid, Julia Fariña, Bernd Rehm, Volker Sieber

**Affiliations:** ^1^Chair of Chemistry of Biogenic Resources, Technische Universität MünchenStraubing, Germany; ^2^Laboratorio de Biotecnología Fúngica, Planta Piloto de Procesos Industriales Microbiológicos-CONICETSan Miguel de Tucumán, Argentina; ^3^Cátedra de Química Biológica, Facultad de Ciencias Exactas y Naturales, Universidad Nacional de CatamarcaSan Fernando del Valle de Catamarca, Argentina; ^4^Institute of Fundamental Sciences, College of Sciences, Massey UniversityPalmerston North, New Zealand; ^5^The MacDiarmid Institute of Advanced Materials and NanotechnologyPalmerston North, New Zealand

**Keywords:** microbial exopolysaccharides, exopolysaccharide biosynthesis, alginate, rare sugars, tailor-made exopolysaccharides, biofilms, imaging and modeling of exopolysaccharides, scleroglucan

The Research topic “Microbial exopolysaccharides from genes to applications” covers 12 articles dealing with the highly diverse class of microbial exopolysaccharides (EPSs). Many bacteria, archaea, yeast, and filamentous fungi are able to produce EPSs under different conditions. These biopolymers significantly differ in monomer composition, substituent decoration, degree and type of branching as well as molecular weight. Therefore, both chemical diversity and functionality of biopolymers is enormous. Their natural roles range from adhesives, to storage compounds, protective hulls, as well as pathogenicity factors. The complete field of putative natural applications is not fully understood up to now. A similar situation is observed for the different biosynthetic pathways. Only minimal information is available for EPS biosynthesis in fungi and similarly, little is known for cyanobacterial and archaeal polysaccharide synthesis routes. In addition to these challenges from the biological point of view, the current methods for polysaccharide analysis are still limited due to many different constraints, which in conjunction make exopolysaccharides a challenging topic of study. One of these limitations is the low achievable EPS concentration often caused by their high viscosity, which, for example, lowers the efficiency of NMR analysis.

Despite, all the remaining challenges and obstacles concerning the study of molecular processes underlying formation of EPS and their chemical characterization, many aspects of this highly diverse class of biopolymers are already known sustaining them as biomolecules of industrial interest.

In the series of articles presented in this book, the authors provide an overview of the different fields involved in microbial EPS production, characterization, and applications. Particular emphasis is directed toward the molecular mechanisms of EPS biosynthesis and modification as well as their regulation. Additionally, the production of fungal EPSs is also explored to show the potential use of these currently less understood microbial biopolymers. Furthermore, the problems and future perspectives related to polysaccharide characterization are summarized and described. Finally, the various aspects of microbial polysaccharide application covering a wide range of uses are also discussed. In summary this research topic deals with the following aspects.

Effective microbial EPS production is based on the identification of novel and efficient production strains. Therefore, Rühmann et al. describe and compare the different available methods to identify EPS producers, including the characterization of monomer composition of these polysaccharides. They finally disclose the most promising and currently available novel techniques, as the main input for boosting the exploration and discovery of new EPS-producers in the short term.

The review of Schmid et al. gives a comprehensive overview of the different biosynthetic pathways known for bacterial EPS-producers and compares in detail the different kinds of EPS. It summarizes the regulatory mechanisms of bacterial EPS production and describes present and future engineering strategies toward tailor-made EPS variants.

The minireview of Becker goes much deeper into this topic, with a specific focus on xanthan and succinoglycan biosynthesis. It includes challenges and perspectives in combinatorial assembly of the biosynthetic pathways in order to obtain tailored variants.

The enhanced bacterial persistence due to enzymatic EPS modifications is described in the review of Whitfield et al. They describe the effect of different enzymatic activities, which are involved in the modification of different EPSs such as alginate, Pel polysaccharide as well as a nitrogen-containing EPS, and link them to their biological function with respect to enhanced survival of the producing microorganisms, either in pure cultures or in biofilms.

The contribution of Ertesvåg especially focuses on alginate modifying enzymes, and discusses how the different modifications influence on material properties of the respective alginate variants. This review represents a comprehensive overview of enzymatic tools suitable for tailoring alginate polysaccharides.

In the original research article of Jachlewski et al. different techniques are presented for the targeted isolation of various EPS and further polymeric compounds, such as DNA and proteins, from biofilms of extremophilic archaea. The authors present a combined approach of proteome and EPS analyses, which provides further insight into the composition and functionality of extremophile biofilms and alludes to the potential of biofilms in future applications.

The special class of microbial EPSs produced by lactic acid bacteria is described in detail by Torino et al. The authors give a comprehensive outlook of either capsular or exopolysaccharides produced by lactobacilli and summarize their traditional and novel applications in food and beverage manufacturing. To complete the landscape, the authors provide a wide description of relevant EPS characteristics and the enzymes involved in their biosynthesis.

The section of rare-sugar-containing EPSs, such as fucose or rhamnose, is described by Roca et al. These polymers are uncommon or at least, rarely identified up to now, and open new frontiers for special applications. In this brief review, EPSs containing rare sugars as well as the respective producing strains are presented, along with the cultivation conditions influencing their monomer pattern. Additionally, the authors focus on their downstream processing and discuss the applications of these special polymers in various fields such as e.g., cosmetics, foodstuff, pharmaceuticals, and biomedical applications.

An overview of current and future biomedical applications of microbial EPSs is given by Moscovici. This article explores the various EPS applications starting from the first tested medical applications, such as the use of dextran as plasma expander, up to the latest innovations in the field, like micro- and nanoparticle-based EPS formulations.

The complete scleroglucan production process, one of the few fungal representative EPSs commercially available, is revisited by Castillo et al. In this comprehensive review, they describe the complete fermentative production process as well as some downstream processing clues which have influence on the final EPS properties. The utilization of non-conventional complex media as efficient carbon-sources for sustainable production of EPS is also outlined and discussed.

Putative future solutions for resolving one of the main obstacles of EPS characterization, i.e., the efficient determination of the EPS structure by NMR analysis, are outlined in the article by Larsen and Engelsen. They present a simple but very efficient combination of NMR spectroscopy and molecular modeling as an efficient EPS analytical tool. This approach might be efficiently used to determine the chemical and three-dimensional EPS structures in fast and reliable way in the near future.

Further, insights into the microbial EPS structure characterization by using novel imaging technologies at various length scales are summarized by Lilledahl et al. The authors present the use of different high-resolution microscopic techniques along with the combination of different approaches in order to support the elucidation of structural features of isolated and secreted EPSs in the cell environment.

In conclusion, these contributions summarize important aspects related to microbial biopolymer research. The technical limitations for analyzing/characterizing microbial EPSs, the urgent need to advance our understanding on EPS biosynthetic pathways, as well as the relevance of producing tailor-made EPSs, are widely explored and discussed employing various representative examples. On the other hand, a range of current and future applications of microbial EPSs is presented, which either already, or in the near future, will contribute to a biobased industry.

In summary, a wide readership with interest in bio-polysaccharides and their promising future is expected to find in this research topic a clear overview assessing the currents gaps in our understanding of EPS while already taking advantage of the current knowledge in the field of microbial polysaccharide research, thus identifying still unmet needs informing future R&D programmes.

## Author contributions

JS initiated the research topic and invited editors JF, BR, and VS. The editorial was written jointly by the editors of the topic.

### Conflict of interest statement

The authors declare that the research was conducted in the absence of any commercial or financial relationships that could be construed as a potential conflict of interest.

